# Pitch contour impairment in congenital amusia: New insights from the Self-paced Audio-visual Contour Task (SACT)

**DOI:** 10.1371/journal.pone.0179252

**Published:** 2017-06-15

**Authors:** Xuejing Lu, Yanan Sun, Hao Tam Ho, William Forde Thompson

**Affiliations:** 1 CAS Key Laboratory of Mental Health, Institute of Psychology, Beijing, China; 2 Department of Psychology, University of Chinese Academy of Sciences, Beijing, China; 3 Department of Psychology, Macquarie University, Sydney, New South Wales, Australia; 4 ARC Centre of Excellence in Cognition and its Disorders, Sydney, New South Wales, Australia; 5 Department of Translational Research on New Technologies in Medicine and Surgery, University of Pisa, Pisa, Pisa, Italy; 6 School of Psychology, University of Sydney, Sydney, New South Wales, Australia; Nanjing University, CHINA

## Abstract

Individuals with congenital amusia usually exhibit impairments in melodic contour processing when asked to compare pairs of melodies that may or may not be identical to one another. However, it is unclear whether the impairment observed in contour processing is caused by an impairment of pitch discrimination, or is a consequence of poor pitch memory. To help resolve this ambiguity, we designed a novel Self-paced Audio-visual Contour Task (SACT) that evaluates sensitivity to contour while placing minimal burden on memory. In this task, participants control the pace of an auditory contour that is simultaneously accompanied by a visual contour, and they are asked to judge whether the two contours are congruent or incongruent. In Experiment 1, melodic contours varying in pitch were presented with a series of dots that varied in spatial height. Amusics exhibited reduced sensitivity to audio-visual congruency in comparison to control participants. To exclude the possibility that the impairment arises from a general deficit in cross-modal mapping, Experiment 2 examined sensitivity to cross-modal mapping for two other auditory dimensions: timbral brightness and loudness. Amusics and controls were significantly more sensitive to large than small contour changes, and to changes in loudness than changes in timbre. However, there were no group differences in cross-modal mapping, suggesting that individuals with congenital amusia can comprehend spatial representations of acoustic information. Taken together, the findings indicate that pitch contour processing in congenital amusia remains impaired even when pitch memory is relatively unburdened.

## Introduction

Congenital amusia is a disorder of music perception that has been the subject of considerable research and theory [1, 2]. Although much remains to be understood about this rare disorder, it is generally agreed that amusic individuals exhibit difficulties in fine-grained pitch perception [3], as revealed by elevated pitch thresholds at a group level compared with non-amusic listeners [4–7]. Despite this difficulty, people with amusia can still name and recognise voices and environmental sounds, and have little difficulty interpreting speech intonation that involves large changes in pitch [1, 8]. In addition, despite evidence that individuals with amusia have poor memory for pitch, there is no evidence they have a general impairment of memory, in that digit spans are comparable in amusics and matched controls [9]. These and other findings led to the hypothesis that the core deficit of congenital amusia is a low-level impairment in fine-grained pitch processing [10].

Subtle impairments beyond music processing have been reported, however. For example, when amusics are presented with the prosodic aspect of spoken sentences in the *absence* of linguistic content (i.e., spoken stimuli that are filtered so as to remove linguistic information while preserving pitch contour), they are significantly worse than control participants at discriminating speech prosody [1]. Given that these stimuli preserved the intonation patterns from spoken sentences, this dissociation challenged the hypothesis that the core deficit of congenital amusia is restricted to fine-grained pitch processing. Thus, Patel et al. (2005) [8] proposed the “*Melodic Contour Deafness Hypothesis*”, which suggests that the principle deficit of congenital amusia lies at a higher level of processing and does not arise from a low-level impairment in pitch processing. Indeed, individuals with congenital amusia exhibit difficulties in melodic contour processing [4, 11], although the source of this difficulty is under debate. Melodic contour refers to the rising and falling pattern of intervals within a melody, and whether adjacent notes are higher or lower than one another [12]. The contour of a melody does not consider the precise size of successive pitch intervals, but represents the direction of pitch changes that occur throughout a sequence [13]. The psychological significance of contour has been underscored by findings that infants are sensitive to pitch contour but not other details of melodies [14], and that adults who hear a novel melody tend to remember its contour but not absolute pitches or precise pitch intervals [15–19]. Thus, successful contour processing depends on the ability to identify the direction of individual pitch changes, and on the ability to retain a succession of pitch changes in memory [18–21].

There are two plausible explanations for the impaired melodic contour processing observed in congenital amusia. One possibility is that amusic individuals have a reduced sensitivity to the direction of pitch change [22]. This reduced sensitivity, in turn, leads to an unstable mental representation of musical pitch [23]. Alternatively, amusic individuals may have difficulty retaining pitch information in memory [6, 24–26] (but see [7]). Tests of contour processing, such as the contour subtest of the Montreal Battery of Evaluation of Amusia (MBEA) [27], typically involve asking listeners to compare two consecutive pitch sequences, which may differ in pitch contour. Thus, performance on the task requires both sensitivity to the direction of pitch changes, and the ability to retain the first sequence in short-term memory until the second sequence is presented for comparison.

To evaluate these two explanations, we developed a Self-paced Audio-visual Contour Task (SACT). The SACT was designed to minimize reliance on short-term memory during the evaluation of melodic contour, and to direct attention to pitch contour in each sequence. As will be described in the *Method* section of Experiment 1, memory load was reduced by presenting a visual contour simultaneously with a melodic contour, with the occurrence of sequential tones self-paced by participants. Melodic contours were displayed as a sequence of large dots varying in spatial height and connected to each other by lines. Thus, instead of holding a melodic sequence in short-term memory and comparing it to a subsequent melodic sequence, participants judged whether concurrent melodic and visual contours were congruent with one another (i.e., online matching). This comparison process also functioned to slow down and enhance the temporal dynamics of contour perception. In short, the task diminished reliance on short-term pitch memory while emphasizing and enhancing the perceptual processing of contour.

The task bears some resemblance to a sight-reading task. However, in standard sight-reading tasks, visual stimuli (notation) are presented in a form that depicts entire musical sequences, which are either presented acoustically for comparison (passive task), or performed by participants (active task). Thus, sight-reading involves encoding visual symbols and either comparing this representation to a delayed auditory stimulus, or mapping the representation to a motor output [28, 29]. In contrast, the SACT allows participants to make “online” comparisons between simultaneously presented auditory and visual contours.

The decision to combine melodic and visual contours was motivated by two lines of evidence that visual representations of melodic contour are natural. One line of evidence comes from cross-modal dimensional interactions at the psychophysical level. For instance, sounds with high frequency (i.e., pitch) or intensity (i.e., loudness) are associated with a higher spatial location (i.e., height) and bright lights or colors (i.e., visual brightness), whereas those with low frequency or intensity are associated with a lower spatial location and dim lights or colors [30–38]. Comparable to non-amusics, amusic individuals are able to represent pitch spatially [23], although this mapping is somewhat less efficient among amusics. Another line of evidence comes from the evidence that untrained listeners can activate visual representations of melodic contour [39] and generate drawings of melodic contours that depict the pattern of ascending and descending pitch changes with considerable accuracy [40]. These findings indicate that there is a strong intermodal association between melodic and visual contours [41].

During the SACT, participants must determine whether each pitch is higher or lower than the immediately preceding pitch, and determine whether this relationship is congruent with the concurrent visual contour. If impaired pitch memory were responsible for poor contour processing in congenital amusia, then participants should perform relatively well on this online matching task, given that the SACT places minimal burden on pitch memory. On the other hand, if amusic individuals are genuinely impaired at processing pitch contour, then they should have difficulty detecting incongruence between melodic and visual contours.

## Experiment 1

### Method

#### Participants

MBEA has been widely used for diagnosing amusia over the past decade. As we were specifically interested in individuals with deficits in pitch processing, we administered the three melodic subtests (Scale, Contour, and Interval) of the MBEA (see also [22]). For each subtest, listeners were presented with pairs of melodies and asked to judge whether they were the same or different. Participants in the pitch-impaired (henceforth “amusic”) group were 14 individuals with composite scores on the three subtests at or below 65 out of 90 (72% correct). Another 14 participants with composite scores above the cut-off score comprised the control group. All participants reported normal hearing and normal or corrected-to-normal vision. None reported any auditory, neurological, or psychiatric disorder. As shown in Table 1, although amusics performed significantly worse than controls in all three melodic tests (all *p* < .001), the two groups were matched in age, gender, years of education, years of musical training, and hours of music listening (all *p* > .10). Written informed consent for participation was obtained from all participants prior to the study. This experimental protocol was approved by Macquarie University Ethics Committee (Ref: 5201200890).

**Table 1:** Participants’ characteristics, mean ± SD correct rates on the melodic subtests of the MBEA, and independent-sample t-test results between two groups in Experiment 1.

	Amusics	Controls	DF	*t*	*p* (2-tailed)
Age	23.22 ± 6.02	25.46 ± 8.39	26	-0.81	.42
Gender	9F/5M	9F/5M			
Years of education	14.21 ± 1.97	14.57 ± 2.31	26	-0.44	.66
Years of musical training	0.36 ± 0.63	1.00 ± 1.40	18.10	-1.57	.14
Hours of music listening daily	2.52 ± 2.96	2.75 ± 2.48	26	-0.23	.82
**MBEA (percentage correct)**					
Scale	0.76 ± 0.07	0.92 ± 0.06	26	-6.08 ***	< .001
Contour	0.68 ± 0.08	0.89 ± 0.05	26	-8.08 ***	< .001
Interval	0.61 ± 0.08	0.86 ± 0.07	26	-9.11 ***	< .001
Composite	0.69 ± 0.04	0.89 ± 0.04	26	-13.24 ***	< .001

DF refers to the degrees of freedom and was corrected if the equal variances assumption was violated.

***: *p* < .001.

#### Stimuli

**Fig 1:**
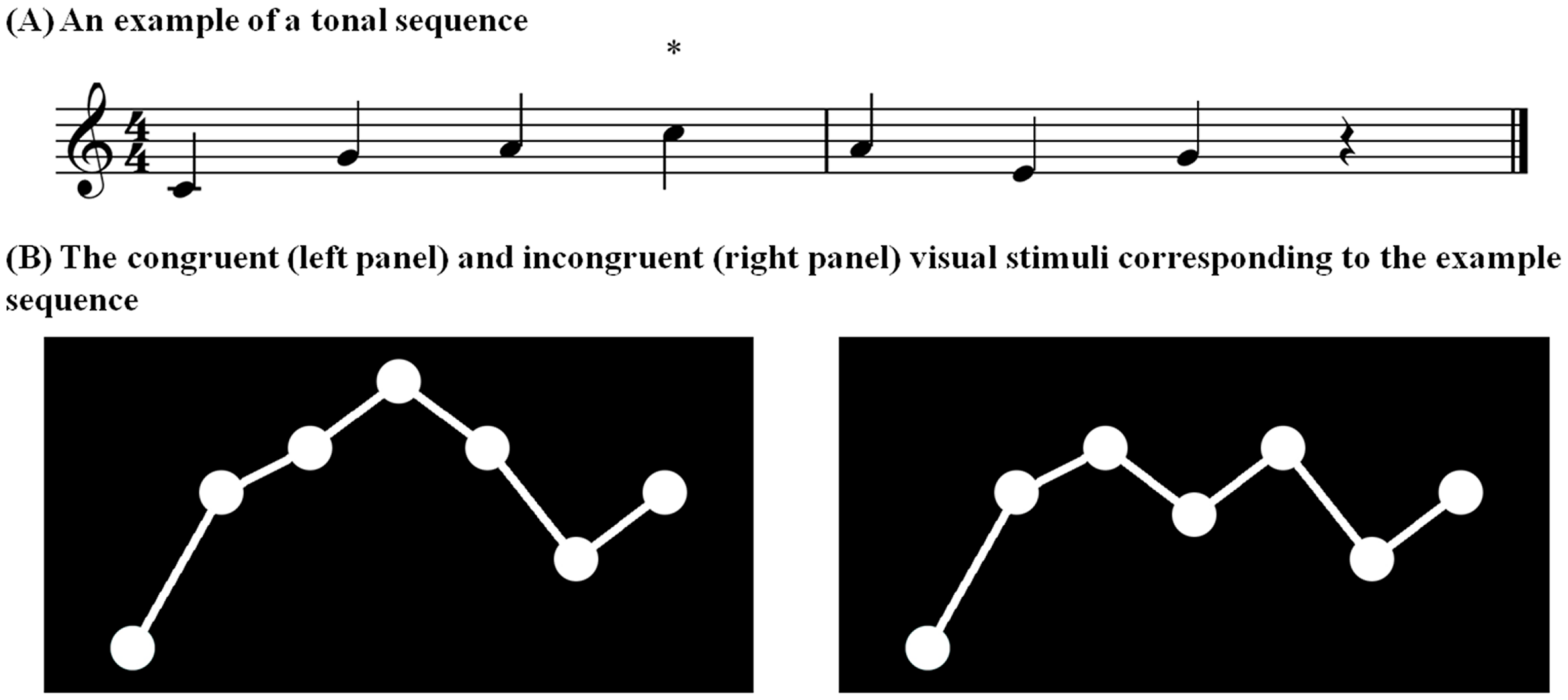
Illustration of the (A) auditory and (B) visual stimuli. * indicates the note that is either congruent (left panel) or incongruent (right panel) with the visual representation. In the incongruent condition, the change in the vertical position of dots is inconsistent with the change in direction of pitches in the accompanying melody.

As illustrated in Fig 1, auditory stimuli were seven-note tone sequences. Each auditory stimulus was accompanied by a visual presentation of a sequence of dots—one for each note of the auditory sequence. The fourth, fifth or sixth tone of each auditory sequence served as a target tone, in which an audio-visual incongruence could occur. The probability of an incongruence occurrence was held constant at each position. Tones in other positions were not selected as targets, because they were used to established a strong sense of tonality. As expected, the target position affected neither amusic and control participants’ task performance (see Figure A in S1 File). The size of the interval separating the preceding and target tones was systematically varied between 1–12 semitones. There were six trials for each interval size condition—half with upward changes and the rest with downward changes. All sequences were constructed using tones from the C major scale, as tonality can boost pitch-related short-term memory in amusics [42, 43]. The strength of tonality of each tone sequence was measured using the “key-finding algorithm” [44] implemented in the MATLAB MIDI toolbox [45], whereby the maximum positive correlation may be taken as the most strongly established key. As expected, the maximum correlation for each tone sequence was with the C major key, *r* (10) = 0.76 (range from 0.45–0.92), all *t* > 3.75, all *p* < .05.

Seventy-two tone sequences were constructed. To ensure that interval size was not confounded with the tonal stability of notes, we monitored the degree of fit (i.e., stability value) of target notes and notes immediately preceding the target notes with respect to the key context (C major in this case). Stimuli were created such that there were no systematic differences across interval sizes in the average tonal stability of tones (all *p* > .05). To ensure results could be generalized beyond a C major key context, a third of the sequences were shifted up by four semitones, and a third of the sequences were shifted down by four semitones. All tones were generated by using the computer software GarageBand (Version 6.0.4; Apple Inc., USA) with flute timbre, and a duration of 500 ms.

Visual stimuli consisted of seven white dots (50-pixel diameter; screen resolution: 1980 × 1024 pixels) that varied in both vertical and horizontal spatial location and that were displayed on a black background and connected by short lines. Each dot corresponded to a single note in the tonal sequence, with pitch height represented as a location on the vertical dimension (a length of 50 pixels in the screen represents a semitone difference), and temporal position represented as a spatial location on the horizontal dimension. The first dot was fixed on the left vertical centre of the display, and the rest were presented successively from left to right simultaneously with the presentation of each tone in the auditory sequence. Two visual stimulus sets were constructed such that melodic and visual contours were either congruent or incongruent with one another.

#### Procedure

Participants were tested in a quiet and dimly lit room. Each trial started with a fixation on the left vertical centre of the screen for 500 ms, at which time the first tone and dot were presented concurrently. Participants were required to push the spacebar on a computer keyboard at a comfortable pace to trigger the following tones and dots, one after another. After the presentation of the whole sequence, participants were asked to make a non-speeded judgment on whether the melodic and visual contours were congruent with one another (“yes” or “no”) by pressing one of two response keys. Assignment of the two response keys for congruent and incongruent trials was counterbalanced across participants. For congruent trials, melodic and visual contours always matched; for incongruent trials, there was a single occurrence within the sequence in which the dot in the visual contour moved in the opposite direction to the direction implied by the pitch change. Twelve practice trials were presented prior to the experimental trials. Feedback was provided during the practice trials but not during the experimental trials. Instead, participants were asked to rate their confidence level for their judgment on a five-point scale (1 = not at all confident; 5 = complete confidence). The congruent and incongruent trials were scrambled independently for each participant, and assigned to six blocks. Participants were encouraged to take a short break after each block to minimize fatigue effects. Auditory stimuli were delivered via noise-cancelling headphones (Sennheiser PXC 350) at a comfortable hearing level of 65 dB SLP. The experiment was programmed and presented in SuperLab 4.5 (Cedrus Corporation, San Pedro, USA).

### Results

For analysis purposes, we classified the interval sizes into three categories: small (1–4 semitones), medium (5–8 semitones) and large (9–12 semitones). This procedure allowed us to reveal broad effects of interval size on the detection of audio-visual incongruence.

The audio-visual judgments were evaluated using d-prime (d′) [46]. A response of “no” on an incongruent trial was defined as a hit whereas a response of “no” on a congruent trial was defined as a false-alarm. A repeated-measures analysis of variance (ANOVA) was conducted with the between-subject factor of Group (amusics and controls) and the within-subject factor of Interval size (small, medium, and large). Greenhouse-Geisser adjustments were used in light of violations of sphericity, and the Bonferroni correction was used in post-hoc analyses, where appropriate.

**Fig 2:**
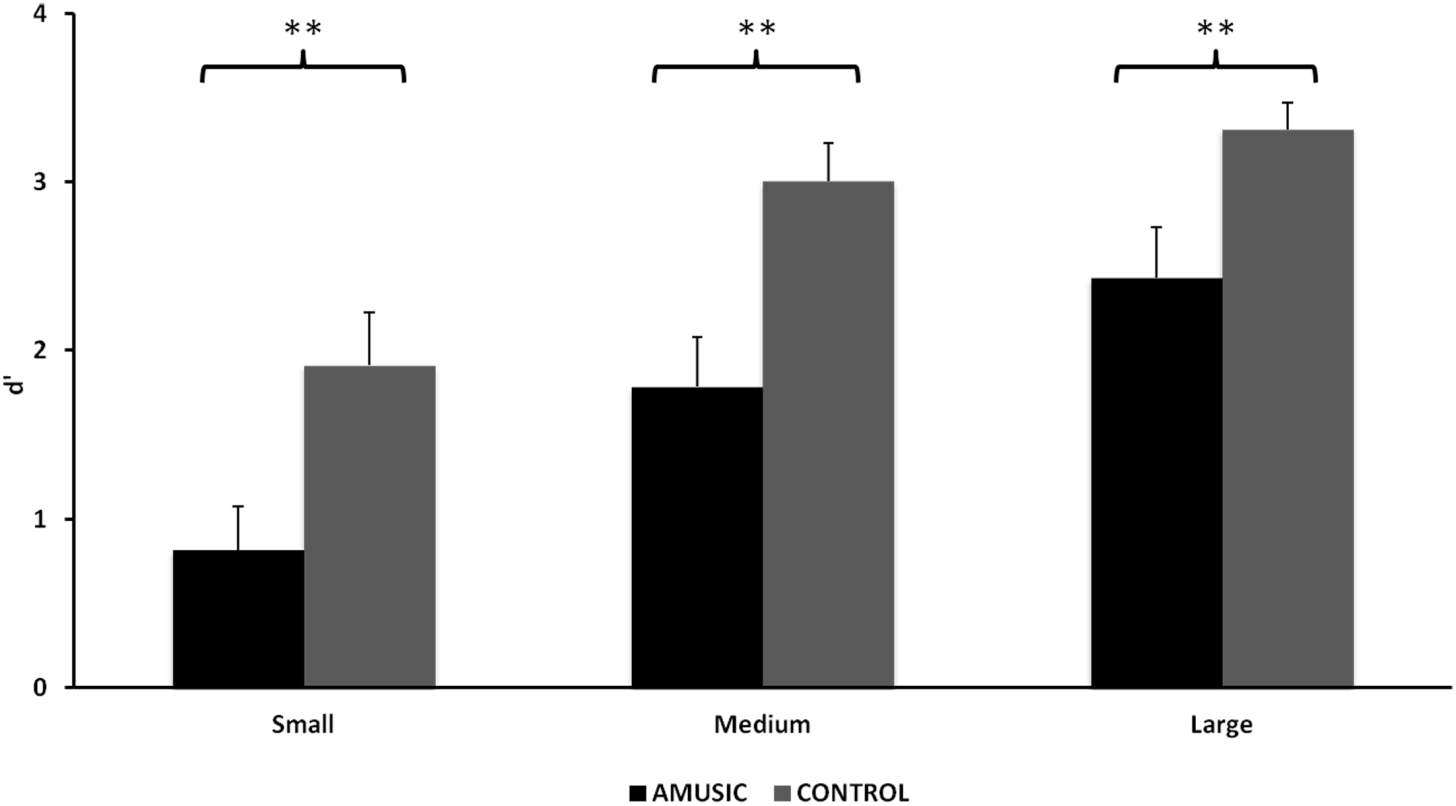
Task performance in Experiment 1 evaluated by d′ on each interval size condition for amusic (black bars) and control (grey bars) groups. Error bars represent +1 SE. **: *p* < .01.

As shown in Fig 2, amusic individuals performed worse than control participants in all interval size conditions, as revealed by a significant main effect of Group, *F*(1, 26) = 11.73, *p* < .01, *η*_*p*_^*2*^ = .31, with no significant interaction with Interval size, *F*(1.59, 41.22) = 0.86, *p* = .41, *η*_*p*_^*2*^ = .03. A main effect of Interval size was also found, *F*(1.59, 41.22) = 72.20, *p* < .001, *η*_*p*_^*2*^ = .70, with increased performance associated with larger interval sizes. It should be noted that the analysis of all 12 interval sizes also yielded significant main effects of Group and Interval size, *F*(1, 30) = 12.05, *p* < .005, *η*_*p*_^*2*^ = .32, and *F*(1, 30) = 29.16, *p* < .001, *η*_*p*_^*2*^ = .53, respectively.

A repeated-measures ANOVA was conducted on the confidence ratings for trials with a correct response, with the factors of Group, Interval size, and Congruence. There was a significant main effect of Interval size, *F*(2, 52) = 44.71, *p* < .001, *η*_*p*_^*2*^ = .63. A post-hoc test indicated that participants’ confidence level increased with the size of the pitch interval leading to the target note (small: *M* = 3.75, *SE* = 0.12; medium: *M* = 3.98, *SE* = 0.11; large: *M* = 4.18, *SE* = 0.10; all *p* < .001). There was also a significant main effect of Group, *F*(1, 26) = 5.03, *p* = .03, *η*_*p*_^*2*^ = .16. Across interval sizes, mean ratings of confidence were lower for individuals with congenital amusia (*M* = 3.78; *SD* = 0.58) than for control participants (*M* = 4.23; *SD* = 0.54). There was no significant interaction involving Group (all *p >* .05). That is, confidence was lower for amusic participants than for control participants regardless of the size of the pitch interval leading to the target note.

### Discussion

These results confirm that amusic participants were less sensitive to audio-visual contour congruence, regardless of the interval size between consecutive tones. Indeed, amusics participants reported low confidence ratings when compared with control listeners for all interval sizes, even when they responded correctly. This deficit is unlikely to be caused by a high threshold for pitch discrimination, because the impairment was observed for small, medium, and large pitch intervals. Pitch memory problems are also unlikely to be the source of the impairment, given that memory requirements in our task were low.

Two possibilities remain. First, the difficulties in detecting incongruence of audio-visual contours may indicate that amusic participants had an unstable spatial representation of pitch. Second, their difficulties may reflect a general impairment in all forms of cross-modal mapping, given that cross-modal mapping is needed to compare stimuli from two sensory modalities. To test the latter possibility and examine whether the impaired contour processing extends beyond the pitch dimension, a follow-up experiment was conducted by employing the same paradigm used in Experiment 1 (i.e., SACT) to investigate contour processing in auditory dimensions other than pitch: either timbral brightness or loudness. Although it has been suggested that temporal, spectral, and intensity perception is intact in the amusic auditory system [47, 48], no study has tested whether individuals with congenital amusia show impairments in contour processing for attributes of sound other than pitch.

## Experiment 2

Although the concept of contour has traditionally been applied to melodies consisting of a sequence of tones that vary in pitch, the contour of acoustic attributes other than pitch also have psychological significance [49, 50], including timbral brightness and loudness. Brightness is one of the most salient dimensions of timbre [51], and reflects the spectral profile of the sound. The perception of brightness correlates with the centre of mass of the frequency spectrum. Sounds with more energy in the high-frequency range of the spectrum are perceived as brighter, whereas sounds with more energy in the low-frequency range are perceived as duller, even when they have the same fundamental frequency (F0). In other words, brightness can be varied independently of the F0 (i.e., pitch height). Loudness, on the other hand, is a non-spectral dimension, and correlates with the intensity of a sound.

If individuals with congenital amusia have contour processing impairments specific to the pitch dimension, then they should be able to discriminate patterns of change in other dimensions. However, if individuals with congenital amusia have a more general impairment in cross-modal mapping, then they should exhibit impaired processing of contours in brightness and loudness.

### Method

#### Participants

Sixteen individuals with congenital amusia (four were recruited from Experiment 1) and 16 matched controls took part in Experiment 2. Table 2 summarizes the participants’ characteristics and results of the MBEA for each group. Because the participant pool and recruitment procedures were identical in Experiments 1 and 2, it is reasonable to assume that the two groups of amusics are representative of the same parent population.

**Table 2:** Participants’ characteristics, mean ± SD correct rates on the subtests of the MBEA, and independent-sample t-test results between two groups in Experiment 2.

	Amusics	Controls	DF	*t*	*p* (2-tailed)
Age	23.34 ± 5.42	23.37 ± 8.73	30	-0.01	.99
Gender	6F/10M	6F/10M			
Years of education	14.38 ± 2.03	13.88 ± 2.33	30	0.65	.52
Years of musical training	0.44 ± 1.09	0.47 ± 0.96	30	-0.07	.94
Hours of music listening daily	2.57 ± 2.15	1.84 ± 0.94	30	1.24	.22
**MBEA (percentage correct)**					
Scale	0.69 ± 0.09	0.92 ± 0.06	30	-8.45 ***	< .001
Contour	0.65 ± 0.08	0.85 ± 0.09	30	-6.29 ***	< .001
Interval	0.66 ± 0.08	0.79 ± 0.08	30	-4.56 ***	< .001
Composite	0.67 ± 0.04	0.85 ± 0.05	30	-12.25 ***	< .001

DF refers to the degrees of freedom and was corrected if the equal variances assumption was violated.

***: *p* < .001.

#### Stimuli

Auditory stimuli were constructed to vary in either timbral brightness or in intensity, but not in pitch. Brightness-varying stimuli were digitally synthesized and manipulated by shifting the spectral centroid while keeping the F0 fixed using MATLAB, following the strategy described by Russo and Thompson (2005) [52] and Warrier and Zatorre (2002) [53]. The energy in the dull timbre was weighted in lower partials, and the energy in the bright timbre was weighted in the higher partials. In the present experiment, F3 (174.62 Hz), G3 (196 Hz), A3 (220 Hz) or B3 (246.94 Hz) was selected to serve as the F0.

**Fig 3:**
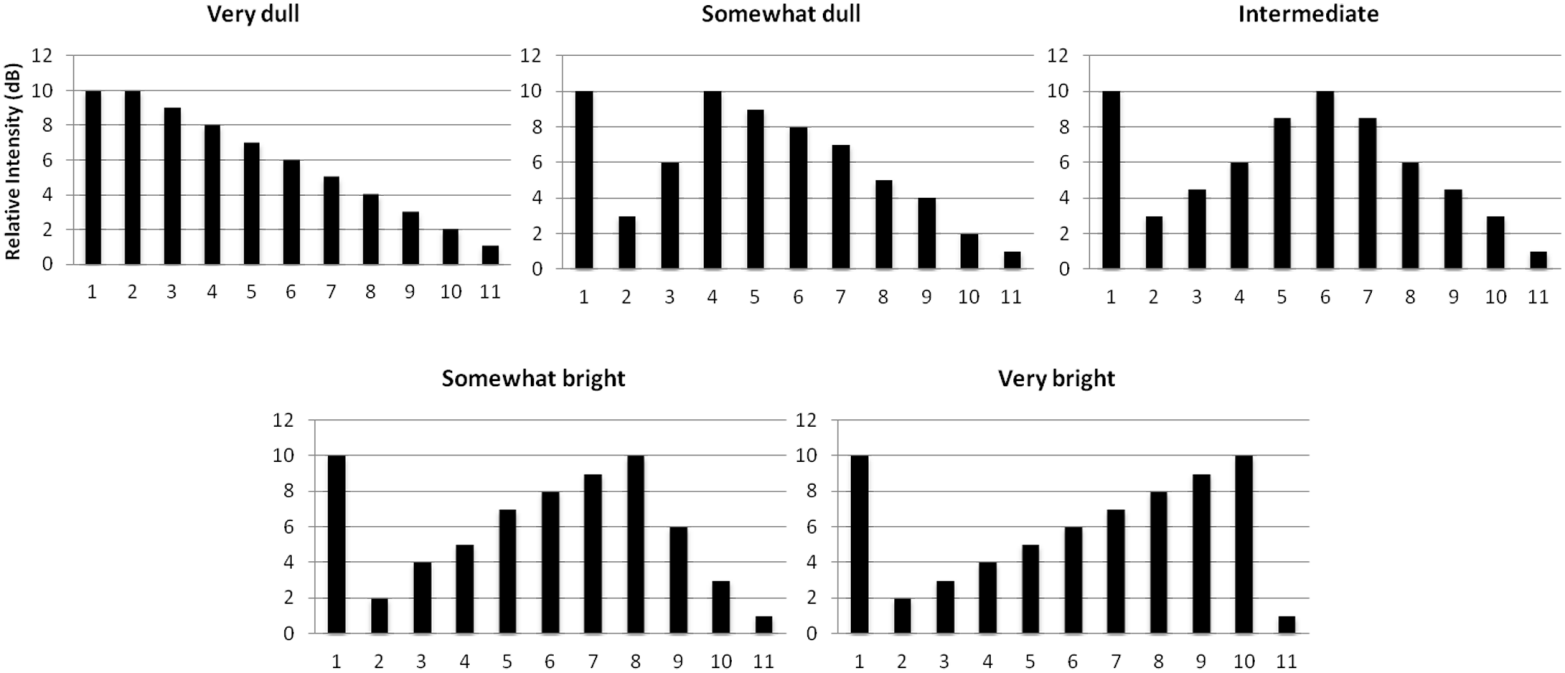
Spectra of five levels of brightness used in Experiment 2. The x-axis refers to the harmonic components.

As illustrated in Fig 3, five levels of timbral brightness (very dull, somewhat dull, intermediate, somewhat bright, and very bright) were generated for each frequency by varying the intensities of ten partial harmonics while keeping F0 at a fixed intensity level that was always higher than that of any other partial. This strategy ensured that tones differed only in brightness but not on the intensity of the F0. The spectral centroid changes from the *intermediate* timbre to the very dull, somewhat dull, somewhat bright, and very bright timbre were approximately 330, 114, 107, and 278 cents, respectively. To be comparable to the pitch-varying sequence used in Experiment 1, timbral brightness-varying sequences consisted of seven notes, and the target note occurred at the fourth, fifth or sixth position at an equal rate. There were 24 timbral brightness-varying sequences in total and the notes in each sequence shared the same F0 but varied in spectral centroid. The degree of change in brightness between the preceding and target note was manipulated experimentally: for half of the trials, small changes in timbre were introduced (a change by one or two levels) and the rest were large changes (a change by three or four levels). Every sequence started with the tone in the intermediate timbre and was presented binaurally at 65 dB SPL through headphones.

The loudness-varying stimuli were generated using the intermediate timbre tones from the timbral brightness-varying stimuli, and manipulated by altering the amplitude to increase or reduce intensity by 5 or 10 dB. Loudness-varying sequences were then created following similar procedures with timbral brightness-varying ones, where the degree of change in loudness between the preceding and target note was either small or large. Thus, loudness-varying sequences varied in intensity, but not in pitch or timbral brightness.

Two types of visual stimuli were employed—height-varying and visual brightness-varying contours. The former stimuli were similar to those used in Experiment 1 and the latter stimuli were created by varying visual brightness while maintaining the vertical spatial location of the dots. In congruent trials, the audio and visual information were congruent; that is, the sound went brighter or louder aurally as the dot moved up or went brighter visually, or the sound went duller or softer aurally as the dot moved down or went dimmer visually. Incongruent trials were characterized by the reversed mapping.

#### Procedure

The paradigm and procedure were the same as that described for Experiment 1. Experiment 2 consisted of four audio-visual combinations: spatial height and timbral brightness; spatial height and loudness; visual brightness and timbral brightness; and visual brightness and loudness. Within each condition there were three blocks of 16 trials, yielding 48 trials per condition. Assignment of the two response keys to the congruent and incongruent responses and the orders of four conditions were counterbalanced across participants.

### Results

**Fig 4:**
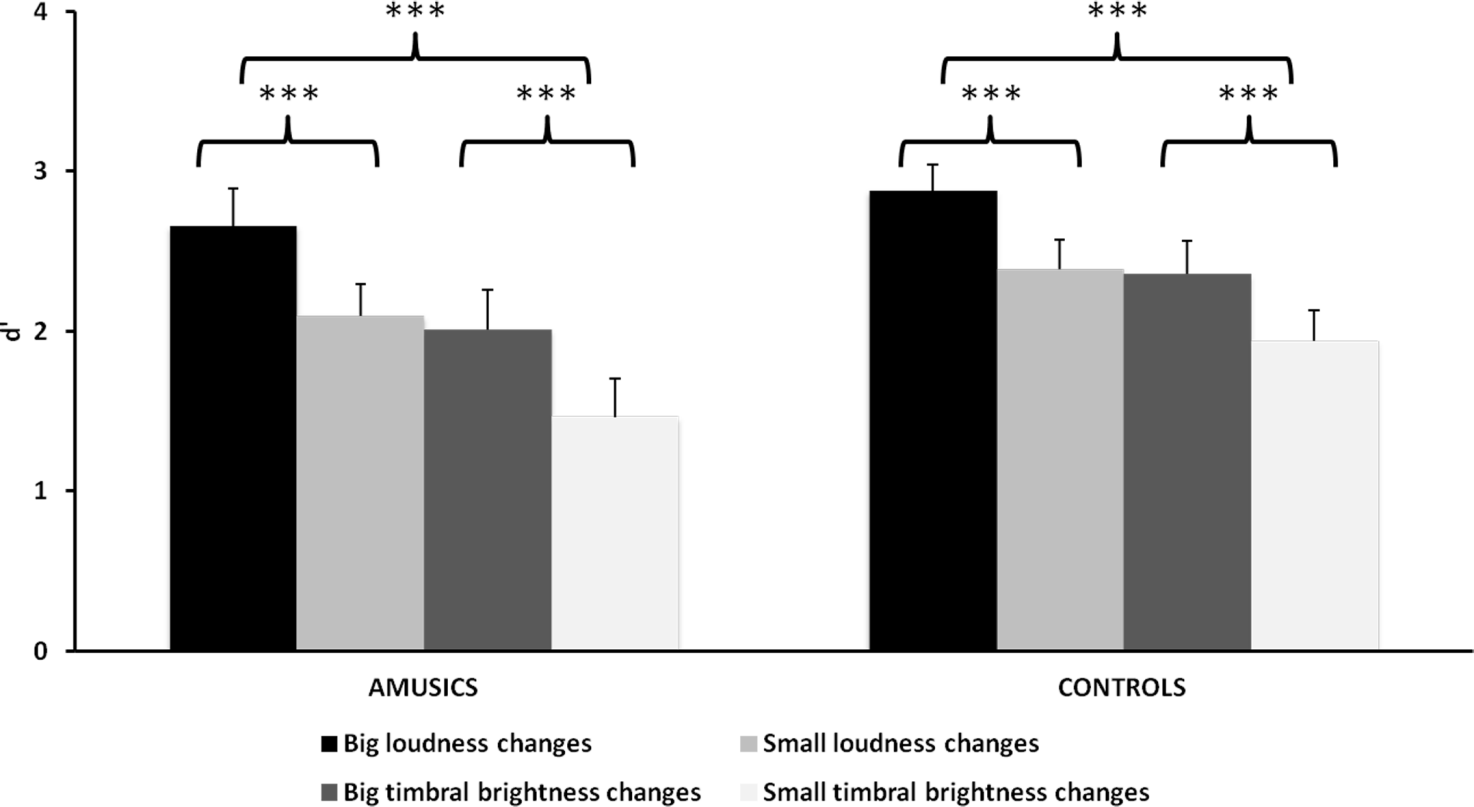
Mean d′ value in Experiment 2 for amusic and control groups. Error bars represent +1 SE. ***: *p* < .001.

A repeated-measures ANOVA on d′ was conducted with the factors of Group, Audio change (timbral brightness and loudness), Visual change (height and visual brightness), and Change size (small and large). As shown in Fig 4, the mean performance in the amusic group (*M* = 2.06, *SE* = 0.16) was slightly lower than the mean performance in the control group (*M* = 2.39, *SE* = 0.16); however, the difference between the two groups was not statistically significant, *F*(1, 30) = 2.11, *p* = .16, *η*_*p*_^*2*^ = .07. All participants did better on loudness-varying conditions (visual height: *M* = 2.52, *SE* = 0.12; visual brightness: *M* = 2.49, *SE* = 0.13) in comparison to brightness-varying conditions (visual height: *M* = 2.03, *SE* = 0.15; visual brightness: *M* = 1.85, *SE* = 0.14), as revealed by the main effect of Audio change, *F*(1, 30) = 34.79, *p* < .001, *η*_*p*_^*2*^ = .54. Similar to the finding in Experiment 1, individuals both with and without congenital amusia showed better performance on trials with large changes (*M* = 2.48, *SE* = 0.12) than on trials with small changes (*M* = 1.97, *SE* = 0.12), *F*(1, 30) = 36.20, *p* < .001, *η*_*p*_^*2*^ = .55.

Similarly, a repeated-measures ANOVA was conducted on confidence ratings. No group difference in self-reported confidence level was found (Amusic: *M* = 4.11, *SE* = 0.13; Control: *M* = 3.88, *SE* = 0.13), *F*(1, 30) = 1.47, *p* = .24, *η*_*p*_^*2*^ = .05. Furthermore, participants reported higher confidence levels on loudness-varying conditions (*M* = 4.15, *SE* = 0.10) when compared with brightness-varying conditions (*M* = 3.83, *SE* = 0.11), *F*(1, 30) = 16.68, *p* < .001, *η*_*p*_^*2*^ = .36, in line with their task performance reflected by d′ value.

### Discussion

These findings suggest that individuals with congenital amusia, who are impaired on tests of melodic contour, have no significant impairment in contour processing for auditory dimensions other than pitch (timbral brightness or loudness in this case). Furthermore, individuals with congenital amusia exhibit no performance deficit for tasks that require cross-modal matching, as long as that task does not rely on sensitivity to pitch contour.

## General discussion

Sensitivity to melodic contour is fundamental to music perception, and impaired contour processing is one of the defining characteristics of congenital amusia. Previous investigations of melodic contour have relied on a discrimination paradigm, in which participants were presented with pairs of melodies and were then required to judge whether they are the same or different (e.g., [4, 11, 25, 27]). However, such an experimental design cannot exclude potential influences from the limited capacity of auditory memory [6, 9, 24]. In this investigation, we developed and employed an online Self-paced Audio-visual Contour Task (SACT) that placed minimal load on auditory memory. The novel task allows us to determine whether the impaired contour processing characteristics of individuals with congenital amusia arises as a secondary consequence of a core deficit in either pitch discrimination or pitch memory. In addition, the self-paced paradigm allows participants sufficient time to encode acoustic information at early stages of the auditory processing, which should optimize pitch processing [43, 54]. Thus, poor performance on the SACT indicates a significant impairment of contour perception that cannot be explained as a consequence of impaired pitch memory.

In Experiment 1, amusic participants exhibited reduced sensitivity to pitch contour, even for pitch interval sizes that exceeded the threshold of pitch discrimination. Given the low memory load in the SACT, this impairment in contour processing cannot be solely explained by deficits in storing and retaining pitch information [6, 24, 42]. In addition, even when amusic participants made correct judgments about pitch contour, they still reported low confidence relative to control, suggesting amusic participants have a cautious attitude towards pitch-related tasks.

The results of Experiment 2 suggest that impaired contour processing is restricted to pitch sequences, and does not extend to acoustic attributes other than pitch. Nor does the impairment reflect a general deficit in cross-modal mapping. Indeed, confidence levels for judgments of timbral brightness and loudness did not differ between participants with and without congenital amusia. That is, amusics can make judgments of auditory input with normal levels of confidence, as long as those judgments are not about pitch [55–57].

What is the underlying mechanism of contour processing in congenital amusia? Our investigation makes three advances to the understanding of impaired contour perception among individuals with congenital amusia. First, given that the SACT exerts very low burden on pitch memory, we can infer that congenital amusia is associated with impaired contour processing, and this deficit is unlikely to be a secondary consequence of an underlying pitch memory problem. Specifically, our results indicate that deficits in contour processing exist independently of potential deficits in short-term memory. Of course, as we did not attempt to isolate later stages of auditory processing (e.g., auditory short-term memory), it is also possible that amusia is independently associated with short-term pitch memory impairments (see [58] for a recent review). Second, given that performance was significantly worse for amusic participants than control participants even when pitch interval sizes exceeded their threshold of pitch discrimination, we can infer that impaired contour processing is unlikely to be a consequence of an inability to *discriminate* successive pitches in melodies. Third, given that amusic and control participants performed equally well on contour matching for acoustic attributes *other* than pitch, our results suggest that impaired contour processing in congenital amusia is specific to the domain of pitch.

Taken together, these insights suggest that impaired contour processing in congenital amusia is not a consequence of poor pitch memory or elevated pitch discrimination thresholds. Two possibilities remain. First, the impairment may reflect unstable coding of pitch *direction* at relatively early stages of processing. This stage of processing would be subsequent to the earliest stage of pitch processing at which individual pitches are encoded. This type of impairment would mean that individuals with amusia can perceive individual pitches, but they have difficulty perceiving relationships between pitches [22]. However, a recent study employing Event-related potential (ERP) methodologies casts doubt on this possibility. Lu et al. (2016) [59] reported that amusic and control individuals exhibited comparable ERP components in response to pitch change directions, suggesting that pitch direction is registered by the amusic brain.

A second possibility is that impaired contour processing in congenital amusia reflects of disorder of pitch awareness. Reduced pitch awareness may arise from an abnormality in the pathways that connect pitch perception to high-level regions associated with conscious awareness [60–62]. This so-called “disconnection hypothesis” predicts that amusic individuals successfully represent pitch change direction at an early stage of processing, but this representation is not reliably transmitted to higher levels associated with conscious awareness, thereby resulting in reduced performance in tasks that entail explicit judgments of pitch [55, 60, 63–65]. A reduction in pitch awareness would also explain why amusic participants were not confident with their pitch judgments, even when those judgments were correct, but they were confident of the judgments that they made for acoustic attributes other than pitch.

## Supporting information

S1 Dataset Raw data underlying the findings. (XLSX)

S1 File The results of additional analyses. (RAR)
